# 分析化学综合实验：电喷雾电离质谱分析双链脱氧核糖核酸和天然药物的非共价相互作用

**DOI:** 10.3724/SP.J.1123.2024.12026

**Published:** 2025-11-08

**Authors:** Lei MA, Yinghui WANG, Yunlan YUAN, Shanshan WANG

**Affiliations:** 长安大学理学院，陕西 西安 710064; School of Sciences，Chang’an University，Xi’an 710064，China

**Keywords:** 分析化学, 实验教学, 质谱分析, 化学教育, 非共价相互作用, analytical chemistry, experimental teaching, mass spectrometry analysis, chemistry education, non-covalent interaction

## Abstract

针对传统分析化学实验课程教学中仪器分析缺失等问题，本研究基于成果导向教育（OBE）理念，提出综合实验的育人目标、素养目标和课程目标。在此背景下，以软电离质谱技术为核心，结合非共价相互作用的教学内容，设计了电喷雾电离质谱（ESI-MS）测定双链脱氧核糖核酸（DNA）和天然药物柚皮苷非共价相互作用的综合实验。该实验面向本科三年级学生，以选修形式开放。实施过程践行线上、线下混合式教学模式，包含课前预习、课中实践和课后复盘环节，搭建了完备的教学平台。实验流程具体包括样品溶液配制、双链DNA的退火合成、药物与DNA反应以及质谱分析和数据处理，体现了多学科交叉知识的综合运用。学生运用ESI-MS负离子和正离子分析模式，结合一级和二级质谱（MS/MS）数据，深入探讨柚皮苷与目标DNA的结合计量比、相对结合强度和作用力类型等信息。实验考评从预习、操作到成果展示，全方位评估学生的综合能力，实行过程性评价，构建了完整的教学体系。本实验将基础知识与科研前沿相融合，操作安全、易行，具有很强的趣味性、拓展性和创新性。该实验的开展不仅丰富了分析化学课程的教学内容，激发学生的科研兴趣，锻炼学生全面的思辨能力，提高其安全意识，还为其未来的科研工作奠定了坚实基础，实现了全面育人的实验教学目标。

## Abstract (en)

Analytical chemistry experiments are essential foundational courses for first- and second-year undergraduates in chemistry， chemical engineering， materials science， and pharmacy. These courses provide students with principles and operational skills of analytical instruments， alongside training in qualitative and quantitative analysis. However， current teaching practices face three main challenges： （1） insufficient focus on instrumental analysis； （2） outdated experimental content misaligned with modern scientific advancements； and （3） limited experimental hours due to curriculum constraints. To systematically address these issues， we proposed educational objectives， competency goals， and course objectives based on outcome-based education （OBE） philosophy. Building upon this backdrop， a comprehensive experiment utilizing electrospray ionization mass spectrometry （ESI-MS） was designed to investigate non-covalent interactions between double-stranded deoxyribonucleic acid （DNA） and naringin， a flavonoid natural drug. The experiment is offered to third-year undergraduate students as an elective. In the implementation process， a blended teaching model combining online and offline methods is adopted. The experimental teaching process is structured into three stages： pre-class preparation， in-class practice， and post-class review. Pre-class tasks include literature reviews， artificial intelligence （AI）-assisted summaries， pre-lab report writing and group discussions. This part is mainly conducted online without occupying class hours. During in-class practice， students synthesized double-stranded DNA by annealing single-stranded DNA （heated at 90 ℃ for 15 min， followed by slowly cooling to （25±1） ℃ overnight and stored at -20 ℃）. The resulting DNA was incubated with naringin at a 1∶4 concentration ratio in ammonium acetate for 15 min. The mixture was then analyzed by ESI-MS on a linear ion trap mass spectrometer. Both negative and positive ion modes were employed with optimized parameters encompassing spray voltage， capillary voltage， tube lens offset， heated capillary temperature， nitrogen sheath and auxiliary gas flows. Data acquisition involved 150 averaged scans using Xcalibur software. ESI-MS under negative ion mode was used to detect the non-covalent complexes. Secondary mass spectrometry （MS/MS） of 5-charged complex ions showed guanine base loss and minimal drug dissociation， indicating strong non-covalent interactions. In positive ion mode， MS yielded lower complex abundance， likely due to charge redistribution during ionization. The results reveal that naringin binds DNA predominantly via *π-π* stacking and hydrogen bonding， with a 1∶1 stoichiometry （relative abundance 60.91%） and a relative binding affinity of 39.20%. Post-class， students were required to process data， write formal lab reports， create presentations for defense， and design a feasible extension experiment. At the same time， a grading system was established for these three phases. The evaluation system emphasizes formative assessment， focusing on aspects such as compliance with experimental procedures， workflow efficiency， safety measures， teamwork， problem-solving skills， and experimental data handling. This multidimensional approach ensures equitable grading and pedagogical validity. This curriculum bridges research and education by introducing MS-based non-covalent interaction analysis into undergraduate curricula. The extended experimental design permits curricular expansion of the course. Some students designed structure-activity relationship （SAR） investigations of flavonoids （e.g.， naringenin vs. naringin）， revealing the role of glycosylation in DNA binding affinity. In addition， students designed a fluorescence quenching spectroscopy experiment， demonstrating interdisciplinary problem-solving skills. It closely aligns with the OBE philosophy， a student-centered framework that fosters innovation. Feedback indicates that 96% of undergraduates perceived significant improvements in their research capabilities and interdisciplinary integration skills. However， some challenges were noted， including students’ initial hesitancy with advanced instrumentation and limited instruments and drugs. We plan to improve teaching in these areas in the future. In conclusion， this OBE-driven experiment successfully modernized analytical chemistry education in mass spectrometry applications by integrating theoretical knowledge with cutting-edge research skills. The project establishes a comprehensive teaching platform and constructs a holistic teaching system， featuring operational safety and accessibility while offering strong demonstrative value in fostering interest， extensibility and innovation. The experiment not only enriches the content of analytical chemistry courses， inspires students’ research interests， and hones their critical thinking abilities， but also enhances their safety awareness and lays a solid foundation for future research endeavors， thus achieving the comprehensive educational goals of experimental teaching.

分析化学实验是高等院校化学、化工、材料、药学等相关专业本科生第一学年和第二学年的必修基础课程^［1］^。该课程旨在帮助学生掌握分析测试设备的基本原理与操作方法，培养其定性定量分析能力。实验设计应激发学生学习兴趣，提高学生动手和思维能力，为知识应用和科研创新提供有力支持^［2］^。然而，目前我校开展的本科生分析化学实验教学存在以下问题：其一，课程设置以化学分析为主，缺失仪器分析实验环节；其二，实验项目多年未更新，内容陈旧，与学生的认知和时代需求脱节；其三，化学类课程总课时有限，教学偏重理论讲解而实验课时长不足，难以实现理想的课程建设目标。为解决上述问题，教研室经过深入教学诊断后，提出面向大学本科高年级学生开展综合实验的教学改革方案。

作为教育改革的主流方向之一，成果导向教育（OBE）理念自1978年提出以来便在美国、英国等国家得到广泛应用^［3］^。该理念强调“以学生为中心”“以学习成果为导向”及“持续改进”，注重培养学生分析复杂问题的能力、团队精神和创新思维^［2， 3］^。基于OBE理念，针对综合实验确立了“安全意识、独立思考、求真务实、笃学创新”的育人目标；以培养学生知识运用、团队协作、数据处理和科研创新能力为素养目标；以分析化学为核心，整合物理化学、基础化学、生物化学等多学科知识为课程目标。据此反向设计方案，最终通过对实践过程和结果的分析，不断改进实验，完成教学闭环。这一改革不仅丰富了分析化学课程体系，突破了传统仪器分析教学中“重理论、轻实践”和“看得到、摸不着”的局限，更推进人才培养提质增效。

非共价作用是普通化学、基础化学和生物化学中关于分子间相互作用力的教学内容，对药物设计和自修复材料设计方面有重要影响^［4］^，作为比较弱的作用力，学生往往难以理解。双链脱氧核糖核酸（DNA）和药物之间的非共价相互作用是近年来的研究热点，其发展使得从基因水平理解某些疾病的发病机理成为可能，并通过设计药物分子结构寻找有效的治疗药物。同时，DNA与药物之间可能存在氢键、*π-π*堆积、静电吸引等非共价作用，是体现该教学内容的良好载体^［5，6］^。为了得到药物分子和核酸作用的详细信息，实验室通常采用溴化乙锭（EB）荧光猝灭方法、紫外光谱法等，但这些方法的药品用量较大，不适合大规模用于本科生实验^［7，8］^。质谱（MS）分析作为现代仪器分析的一项重要手段，具有药品用量少、快速、灵敏度高的检测优势^［9］^。学生在高中化学和大学分析化学中均已深入学习其原理。此外，了解和掌握质谱分析技术不仅有助于提高基础知识的应用能力，还可以为以后的科研工作奠定基础。经查文献，质谱教学主要偏重定量分析和结构鉴定，用于非共价相互作用的教学实践鲜有报道^［10，11］^。因此，将课程内容和前期科研成果进行科教融合，通过整合电喷雾电离质谱分析和非共价相互作用等多个基础课程教学知识点、设计跨课程体系的综合实验是必要且可行的。

## 1 实验部分

### 1.1 实验目的

（1）巩固基础化学类课程中关于非共价相互作用的理论认知，并强化仪器分析相关专业知识；（2）掌握溶液配制、移液枪使用等基本单元操作；（3）掌握双链DNA退火合成方法和实验步骤；（4）掌握电喷雾电离质谱（ESI-MS）的基本原理和使用方法，掌握实验数据提取和处理方法；（5）利用实验结果分析药物和双链DNA非共价相互作用类型和作用力大小。

### 1.2 实验原理

ESI-MS属于软电离技术，可以较好地保留生物大分子样品的完整性^［12］^。对于双链DNA及其药物复合物，质谱图中通常可以观察到失去多个质子（负离子模式）或捕获多个质子（正离子模式）的多电荷准分子离子峰。多电荷离子的存在，显著扩展了质谱仪的检测范围。通过样品的实际分子质量及其在质谱图中的质量电荷比（*m/z*）信息，可以准确识别对应的离子。根据文献报道，负离子状态下，可以直观并快速地检测到药物和DNA复合物离子，并通过谱图中最高丰度对应的复合物离子确定结合计量比^［12］^。此外，利用公式（1）计算得到相对结合强度，该结果与其他分析方法得到的数据一致。为进一步探索药物与DNA的结合方式，将复合物离子在二级质谱（MS/MS）中的裂解特征与米托蒽醌、道诺霉素等经典药物的碎裂结果进行对比，从而获得结合模式信息^［13-15］^。


$$F=\left(I_{1:1}+I_{1:2}+\ldots \right)/\left(I_{DNA}+I_{1:1}+I_{1:2}+\ldots \right)$$ （1）

式中，*F*表示药物与核酸的相对结合强度，*I*_DNA_表示核酸的相对离子丰度，*I*_1∶_ *_n_* 表示非共价复合物的相对离子丰度，*n*为正整数。

实验选用长度为14 bp的合成双链DNA分子。一方面其分子质量较小，在检测范围内（*m/z* 800~2 000）可观察到DNA及其复合物带5个和6个电荷的准分子离子峰。另一方面，12 bp以上的双链DNA在质谱中更加稳定^［16］^。该目标DNA序列位于存活素基因启动子Sp-1区域-158~-145处，在存活素基因转录中具有重要作用，可促进学生对DNA靶点治疗的认识^［5］^。在众多药物中，天然成分是筛选DNA抑制剂的重要来源^［17］^，引入常见的黄酮类化合物柚皮苷可有效提升实验趣味性。

### 1.3 仪器与试剂

LTQ^TM^ XL线性离子阱质谱仪（配ESI源）和移液枪（美国Thermo公司）；恒温水浴锅（HH-S1，江苏金怡）；1.5 mL塑料离心管（美国Sigma公司）；水浴锅泡沫浮漂。

单链核酸（S1：5′-AACTCCCGGCACAC-3′；S2：5′-GTGTGCCGGGAGTT-3′）由大连宝生物工程有限公司合成；柚皮苷标准品（*M*_w_=580.53）购于中国药品与生物制品检验所；醋酸铵购于瑞士Fluka公司；甲醇（色谱纯）购于美国Fisher公司；实验用水采用美国Millipore超纯水系统；其余试剂均为分析纯。

### 1.4 实验内容

#### 1.4.1 溶液的配制

柚皮苷标准品用甲醇配成浓度为1 mmol/L的储备液，避光保存。

单链核酸样品置于-80 ℃冰箱保存。将单链DNA溶于水，配制成2 mmol/L的储备液。

#### 1.4.2 DNA退火合成及与药物反应

退火合成：取两种单链核酸储备液各50 μL，与100 μL的1 mol/L醋酸铵溶液混合于离心管中。将离心管放置于水浴锅泡沫浮漂，90 ℃恒温水浴保持15 min，缓慢冷却至室温（25±1） ℃，过夜，得到500 μmol/L双链DNA，置于-20 ℃冰箱保存备用。

DNA与药物反应：取1 μL退火合成的双链DNA和2 μL柚皮苷储备液于离心管混合，置于79 μL 20 mmol/L醋酸铵溶液室温下反应15 min。进样前加入18 μL甲醇，最终得到DNA浓度为5 μmol/L的待测样品用于质谱分析。除了不加药物外，采取同样的条件制备DNA待测样品。

#### 1.4.3 质谱分析过程

质谱分析按照进样-测试-数据采集-清洗仪器4个步骤完成。

进样：样品溶液以5 μL/min的流速泵入质谱仪进行测定。

测试：在负离子模式下，设置加热毛细管温度250 ℃，壳气和辅气流速分别为30 units/min和3 units/min，喷雾电压为-3.0 kV，毛细管电压为-22 V，管透镜电压为-130 V；正离子模式下，加热毛细管温度为255 ℃，壳气流速为35 units/min，喷雾电压3.5 kV，毛细管电压和管透镜电压分别为65 V和160 V。二级质谱实验在碰撞诱导解离（CID）模式下进行，设置碰撞能量12%~28%和碰撞时间30 ms。

数据采集：待总离子流稳定后，用Thermo公司Xcalibur软件采集150次平均扫描图谱。

清洗仪器：测试结束后用进样器吸取100 μL甲醇-水（20∶80，体积比）进行洗针，同时采用该溶液重复进样测试步骤3次清洗质谱仪。

#### 1.4.4 数据处理

利用Xcalibur、Office等软件进行数据处理。

## 2 结果与讨论

### 2.1 负离子模式下的一级质谱结果

在负离子模式下，双链DNA的一级质谱结果如图1a所示。学生通过计算质荷比进行特定离子的归属，对谱图进行解析。主要观察到两种离子：第一种为单链DNA离子，即两条单链核酸S1、S2分别带3个和4个负电荷的准分子离子峰；第二种是双链DNA离子，具体为带5个和6个负电荷的双链核酸准分子离子峰，对应*m/z* 1 703.43和*m/z* 1 419.39。其中，带5个电荷的离子相对丰度为100%。

**图1:**
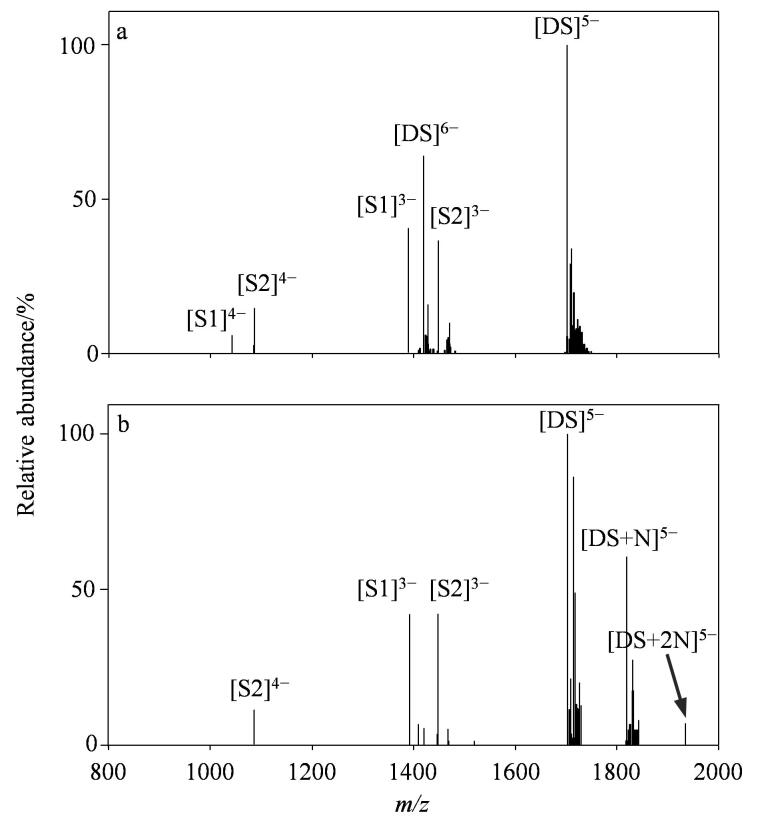
负离子模式下（a）双链DNA和（b）双链DNA与柚皮苷相互作用的质谱图

对于含有柚皮苷和双链DNA的样品，图1b显示存在双链DNA与药物非共价复合物的多电荷离子。其中，以双链DNA结合一个药物分子的离子［DS+N］^5-^为主，即*m/z* 1 819.44，相对丰度为60.91%。同时还形成了核酸与药物分子结合计量比为1∶2的离子［DS+2N］^5-^，其相对丰度更低。以上结果表明柚皮苷和DNA的结合计量比以1∶1为主。

基于质谱分析原理，假设化合物的浓度与质谱中的离子强度成正比^［14］^。可利用公式（1）计算柚皮苷与双链DNA的相对结合强度，实现定量表征，数值越高说明结合力越强。在负离子模式下，选取带5个负电荷的离子进行计算。结果显示，柚皮苷的相对亲和力为39.20%。在确保仪器安全的前提下，学生可自行调整仪器参数，观察不同参数对实验结果的影响。

### 2.2 负离子模式下的二级质谱结果

相比于一级质谱，串联质谱能够提供更多的信息。在负离子模式下，对母离子［DS+N］^5-^（*m/z* 1 819.40）进行二级质谱分析（如图2a所示），未检测到单链核酸离子碎片。主要产生以G碱基丢失为主的子离子［DS+N-GH］^5-^（*m/z* 1 789.43），伴随少量中性药物丢失，最终得到自由核酸离子［DS］^5-^。这种碎裂模式与文献中报道的插入剂道诺霉素类似^［14，15］^。复合物倾向于共价键断裂而不是非共价键分离，表明柚皮苷和DNA结合能力较强，也说明多个弱的非共价作用力的强度大于一个共价键的强度。图2b展示了对带6个负电荷的复合物离子［DS+N］^6-^（*m/z* 1 516.89）进行二级质谱分析的结果，碎裂后主要生成药物配体离子［N］^-^和DNA离子［DS］^5-^。因此，综合考虑柚皮苷与DNA靶分子复合物离子的碎裂规律，推测柚皮苷的母核可通过*π-π*相互作用平行插入DNA分子的双螺旋结构中，与DNA的碱基对形成碱基堆积非共价相互作用。

**图2:**
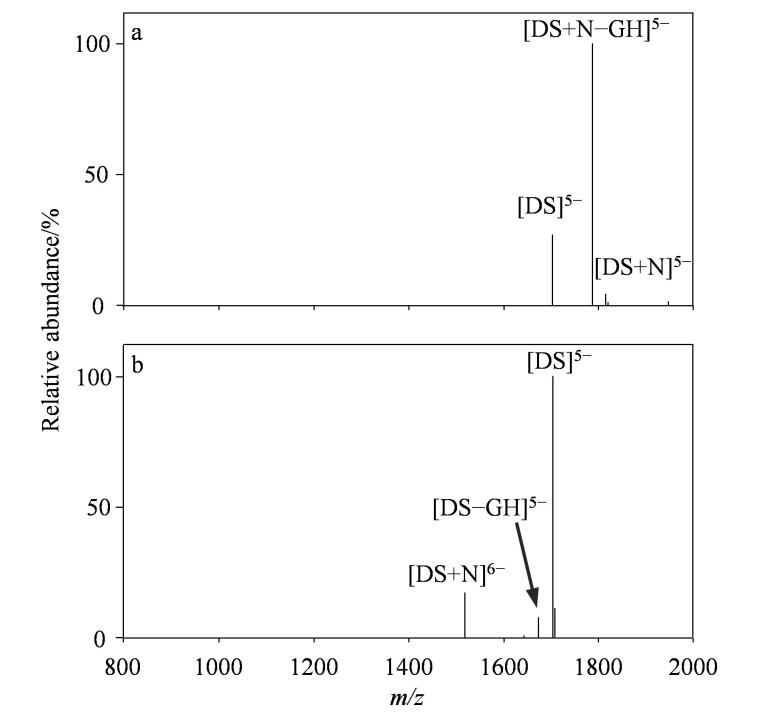
负离子模式下双链DNA与柚皮苷复合物离子（a）［DS+N］^5-^和（b）［DS+ N］^6-^的二级质谱图

### 2.3 正离子质谱结果

在一级正离子质谱全扫描过程中，观察到柚皮苷和双链DNA以1∶1结合的复合物，对应离子*m/z* 1 865.52（如图3所示）。结果说明柚皮苷与DNA之间除了碱基堆积作用还存在氢键结合力^［14］^。推测糖苷中糖羟基（-OH）可能与DNA骨架磷酸基团形成氢键，从而增强与双链DNA的结合。与负离子模式相比，复合物离子在正离子质谱中的相对丰度更低，与道诺霉素等药物的表现一致^［14］^。

**图3:**
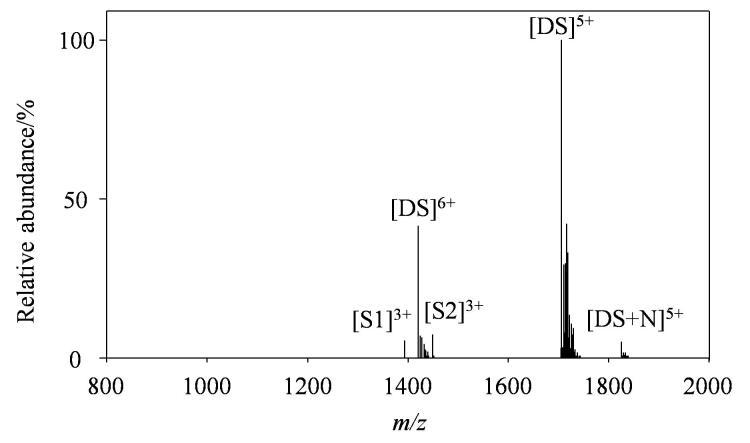
正离子模式下双链DNA与柚皮苷相互作用的质谱图

## 3 实验的实施过程

### 3.1 师生研讨

实验中及实验后，学生与老师围绕实验内容和现象展开深入探讨，重点内容如表1所示。

**表1:** 主要研讨内容

No.	Question	Interactivity
1	Students ask： Why is it necessary to anneal DNA to room temperature slowly？	Teachers answer： The slow cooling process after heating is essential for the proper formation of new hydrogen bonds， ensuring the accuracy and stability of the DNA molecular structure. Teachers guide： Interested students are encouraged to conduct experiments comparing the differences between double-stranded DNA obtained through slow annealing and that obtained through rapid cooling.
2	Students ask： Why is the relative ion abundance of the complex with six negative charges observed to be lower than that of the complex with five negative charges in the negative ion mass spectra？	Teachers answer： The relatively low abundance of 6-charged ions may be due to their additional negative charge compared to 5-charged ions， which can make the ionic state less stable. Teachers guide： Additionally， guide students to check if they observe complex ions between naringin and single-stranded DNA. This is possibly due to the drug's preference for binding more readily to double-stranded DNA.
3	Teachers ask： What are the differences between the positive ion spectrum and the negative ion spectrum？	Students answer： In positive ion mode， the relative abundance of complex ions is lower. The reason for this phenomenon may be related to different processes of electrospray ionization. Specifically， in positive ion mode， the phosphate group is electrically neutral， and the additional positive charge tends to be located in the base pairs within the small groove region of DNA. When drug molecules bind to DNA， they need to disrupt the interaction between the base and NH_4_^+^ and non-covalently bind to the base pair. During this process of breaking the original binding， the positive charge is reduced， leading to fewer complex ions.
4	Teachers guide： Guide students to consider the relationship between the principle of “like dissolves like” and non-covalent interaction.	Students answer： When we use products such as soap and facial cleanser with high concentration， the hydrophilic groups interact with water molecules， while the hydrophobic groups， due to their incompatibility with water， form spherical aggregates and micelles through van der Waals forces.
5	Teachers guide： Guide students to identify examples of non-covalent forces， as this will be very helpful in deepening the application of these concepts.	Students answer： The high boiling point of hydrofluoric acid is due to intermolecular hydrogen bonding. Students answer： The binding of enzymes and substrates also involves non-covalent interactions.

### 3.2 教学设计

基于OBE理念所提出的育人目标、达标素养目标和课程目标，设计了以非共价相互作用为主题的质谱分析综合实验。该实验面向我校大学三年级学生，以选修形式开放。选课学生需满足以下两个基本条件：已经通过我校化学类基础课程学习，掌握双链DNA的基本结构、质谱仪构造和原理等理论知识；通过实验室安全教育与准入考试，完成分析化学实验或普通化学实验且成绩达到80分以上，具备一般化学实验操作能力。教学过程包括课前熟悉、课中实践、课后复盘3个阶段，其总体设计框架如图4所示。

**图4:**
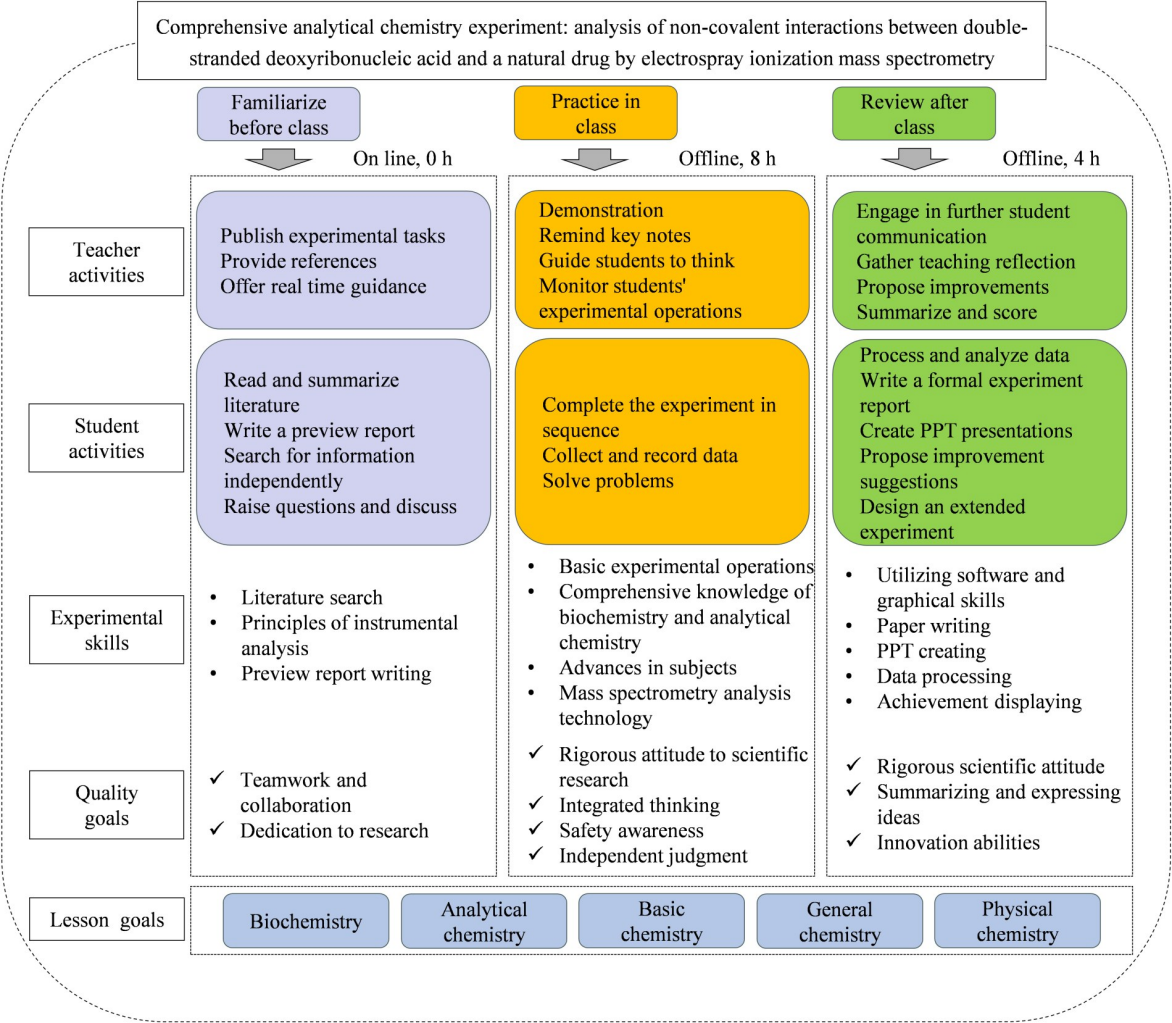
教学设计框架

实验设计2人一组，开设学时12 h。具体实施安排如下。

课前熟悉：教师通过在线教学平台发布实验任务，根据选课情况提前2周提供相关学习资料。要求学生单独提出3个问题并互相讨论，鼓励同学在中国知网等专业网站自行查找文献学习，或利用AI技术撰写文献综述，以提升独立思考能力。学生通过复习以往基础课知识，了解实验原理和环节，制订实验方案和分工，结果以预习报告形式提交。该阶段利用课余时间线上完成，不占学时。

课中实践：老师利用PPT仔细讲解并演示仪器操作，提示学生操作要点，实验中对学生操作进行评判。学生按照既定方案依次完成实验实践。要求学生在实验中遵守操作规程，注意安全防护并如实记录实验数据，培养安全意识和学术诚信品质。需要注意的是溶液配制和DNA退火需要在质谱分析前至少一天完成。如果仪器有限或选课学生较多，建议实验操作部分错峰进行。该阶段共需要8个学时。

课后复盘：实验后，学生利用Origin、Xcalibur等软件处理数据并分析实验结果，积极深入讨论，撰写正式实验报告并制作PPT进行答辩。答辩内容除了质谱实验外，还要求同学设计一个可行的拓展实验，为以后科研探索奠定基础。老师对正式实验报告进行批改，整理打分。另外，师生共同制定改进方案，为后期完善教学提供方向。该阶段共需要4个学时（不含开展拓展实验用时）。

### 3.3 实验考评

实验考评是评判培养目标达成度的重要过程，包括实验预习、实验过程和成果展示3个环节，具体如表2所示。从实验操作合规、流畅度、安全防护、团队合作精神、解决问题能力、实验数据处理情况等方面对学生进行综合评分，保证了实验成绩公平、考查方向全面。

**表2:** 实验考评内容

Assessment process	Assessment criteria	Score proportion/%
Preparation work	（1） acquire a thorough understanding of the binding mode in DNA-drug interactions （5 points）； （2） master the principles of mass spectrometry and other analytical methods （5 points）； （3） submit an experimental preview report with comprehensive experimental steps，well-defined and reasonable personnel roles （5 points）.	15
Experimental process	（1） adherence to standardized experimental procedures （20 points）； （2） adherence to laboratory safety regulations and ensure adequate personal protection （5 points）； （3） proficiency in setting and adjusting experimental parameters （5 points）； （4） record experimental data accurately and truthfully （5 points）； （5） effective teamwork （5 points）.	40
Achievement exhibition	（1） correct experimental data processing and analysis， with clear discussions （10 points）； （2） strict adherence to standard formatting for formal experimental reports （5 points）； （3） create well-prepared PPT presentations （5 points）； （4） deliver a smooth presentation with accurate responses to questions （15 points）； （5） a well-planned and thoughtful extended experiment design （10 points）.	45

## 4 教学效果与反思

本实验教学取得了良好的教学效果，学生掌握了双链DNA退火合成、质谱分析的基本原理、操作流程，对非共价作用力有了深入的理解。经过学生反馈和总结，实验教学具有以下几个特点。

（1）实验设计具有自主化程度高、拓展性强的特点。以本实验抛砖引玉，拓展实验设计，留给学生更多的创造空间，其目的一方面增加实验连续性，另一方面以“未知”刺激学生的探索欲望，发挥学生的主观能动性，培养学生综合运用跨学科知识的能力。某一组同学开展了药品拓展实验，根据公式（1）计算得到了多种药物分子和靶点DNA的相对结合强度，具体数据见图5。由图可以看出，黄酮苷与其苷元相比具有更强的双链DNA结合能力，比如相对结合强度柚皮苷大于柚皮素，甘草苷大于甘草素，黄芩苷大于黄芩素。然而结合强度并非简单地由分子内各部分亲和力相加，比如槲皮苷和槲皮素同属于黄酮醇类化合物，具有一样的母核结构，但取代位的糖配基未对结合力产生显著影响。该结果也说明双链核酸与药物的作用与黄酮类药物母核结构密切相关。尽管黄酮类化合物与DNA之间的精确结合位点并未通过拓展实验确定，但是基于实验结果补充构效关系，起到加强实验深度的效果。

**图5:**
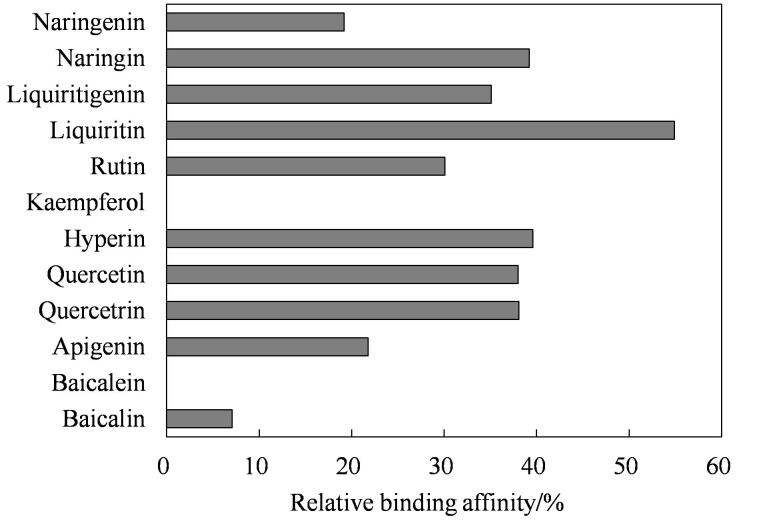
双链DNA与黄酮类化合物的相对结合强度

某一组同学进行了检测方法拓展，选择了荧光光谱分析。实验前考虑到实验成本，将合成DNA替换成小牛胸腺DNA。实验中学生佩戴防护面具，培养了良好的安全意识。实验结果利用经典Stern-Volmer方程^［18，19］^，计算得到静态猝灭常数*K*_A_为7.62×10^3^ L/mol，结合比为0.84，接近1∶1。说明柚皮苷和双链DNA形成了稳定的复合物，与质谱分析数据一致。

（2）学生实验操作能力显著提升。实践表明，学生可以在设定学时内顺利完成实验。对比分析发现，在初次实验过程中存在的药品称量记录缺失等操作问题，在拓展实验中得到明显改善，达到了预期的教学效果。

（3）学生独立科研能力培养效果明显。本实验流程与科研过程一致，利于学生掌握系统的科研方法。经过调研，96%的学生认为该实验“提升科研能力”“加深知识的融会贯通”。基于实验表现选拔的优秀学生，近两年参加学科竞赛取得了10多项荣誉，实现了基础教育与创新人才培养的衔接。

（4）持续优化教学方案。为进一步提高人才培养质量，团队将从以下方面继续探索。优化教学方式：根据所使用的仪器拍摄详细的教学视频，以帮助学生加快理解实验和熟悉仪器操作；拓展教学维度：扩大仪器和药品使用范围，通过多种技术联合验证相互作用力，引导学生深入分析构效关系，加强实验深度和广度。

## 5 结语

实验内容有机整合了分析化学、生物化学等多学科知识，学生通过合成核酸、质谱分析过程，最终直观地看到药物和双链DNA的复合物，将传统教学中抽象的概念具象化，帮助学生在学习新技术的同时加强对理论知识的理解。通过该综合实验的开设，有效促进了学生对现代仪器分析原理与方法的掌握，提升了实验能力、数据分析能力和团队合作能力，加强了动手能力，培养了创新意识，激发了学习热情，符合现代分析化学实验的教学目标。团队将继续从教学方式和教学维度方面改进实验，助力提升人才培养质量和学科发展。
